# Increasing the etanercept dose in a treat-to-target approach in juvenile idiopathic arthritis: does it help to reach the target? A post-hoc analysis of the BeSt for Kids randomised clinical trial

**DOI:** 10.1186/s12969-024-00989-x

**Published:** 2024-05-10

**Authors:** Bastiaan T. van Dijk, Sytske Anne Bergstra, J. Merlijn van den Berg, Dieneke Schonenberg-Meinema, Lisette W.A. van Suijlekom-Smit, Marion A.J. van Rossum, Yvonne Koopman-Keemink, Rebecca ten Cate, Cornelia F. Allaart, Daniëlle M.C. Brinkman, Petra C.E. Hissink Muller

**Affiliations:** 1 Department of Paediatrics - division of Paediatric Rheumatology, Willem-Alexander Children’s Hospital, Leiden University Medical Centre, Leiden, the Netherlands; 2 Department of Rheumatology, Leiden University Medical Centre, Leiden, the Netherlands; 3 Department of Paediatric Immunology, Rheumatology and Infectious Diseases, Emma Children’s Hospital / Amsterdam University Medical Centres, Amsterdam, the Netherlands; 4 Department of Paediatric Rheumatology, Sophia Children’s Hospital, Erasmus Medical Centre, Rotterdam, the Netherlands; 5 Department of Paediatrics, Emma Children’s Hospital / Amsterdam University Medical Centres, Amsterdam, the Netherlands; 6 Department of Paediatric Rheumatology, Amsterdam Rheumatology and Immunology Centre (Reade), Amsterdam, the Netherlands; 7 Department of Paediatrics, Juliana Children’s Hospital / HagaZiekenhuis, the Hague, the Netherlands

**Keywords:** Juvenile idiopathic arthritis, Etanercept, Dose, Treat-to-target, Randomised clinical trial, Efficacy, Adverse events

## Abstract

**Background:**

Etanercept has been studied in doses up to 0.8 mg/kg/week (max 50 mg/week) in juvenile idiopathic arthritis (JIA) patients. In clinical practice higher doses are used off-label, but evidence regarding the relation with outcomes is lacking. We describe the clinical course of JIA-patients receiving high-dose etanercept (1.6 mg/kg/week; max 50 mg/week) in the BeSt for Kids trial.

**Methods:**

92 patients with oligoarticular JIA, RF-negative polyarticular JIA or juvenile psoriatic arthritis were randomised across three treat-to-target arms: (1) sequential DMARD-monotherapy (sulfasalazine or methotrexate (MTX)), (2) combination-therapy MTX + 6 weeks prednisolone and (3) combination therapy MTX + etanercept. In any treatment-arm, patients could eventually escalate to high-dose etanercept alongside MTX 10mg/m^2^/week.

**Results:**

32 patients received high-dose etanercept (69% female, median age 6 years (IQR 4–10), median 10 months (7–16) from baseline). Median follow-up was 24.6 months. Most clinical parameters improved within 3 months after dose-increase: median JADAS10 from 7.2 to 2.8 (*p* = 0.008), VAS-physician from 12 to 4 (*p* = 0.022), VAS-patient/parent from 38.5 to 13 (*p* = 0.003), number of active joints from 2 to 0.5 (*p* = 0.12) and VAS-pain from 35.5 to 15 (*p* = 0.030). Functional impairments (CHAQ-score) improved more gradually and ESR remained stable. A comparable pattern was observed in 11 patients (73% girls, median age 8 (IQR 6–9)) who did not receive high-dose etanercept despite eligibility (comparison group). In both groups, 56% reached inactive disease at 6 months. No severe adverse events (SAEs) occurred after etanercept dose-increase. In the comparison group, 2 SAEs consisting of hospital admission occurred. Rates of non-severe AEs per subsequent patient year follow-up were 2.27 in the high-dose and 1.43 in the comparison group.

**Conclusions:**

Escalation to high-dose etanercept in JIA-patients who were treated to target was generally followed by meaningful clinical improvement. However, similar improvements were observed in a smaller comparison group who did not escalate to high-dose etanercept. No SAEs were seen after escalation to high-dose etanercept. The division into the high-dose and comparison groups was not randomised, which is a potential source of bias. We advocate larger, randomised studies of high versus regular dose etanercept to provide high level evidence on efficacy and safety.

**Trial registration:**

Dutch Trial Register; NTR1574; 3 December 2008; https://onderzoekmetmensen.nl/en/trial/26585.

**Supplementary Information:**

The online version contains supplementary material available at 10.1186/s12969-024-00989-x.

## Background

Pharmacological treatment of non-systemic juvenile idiopathic arthritis (JIA) has undergone substantial transformations during the past two decades [1]. Early initiation of conventional synthetic disease-modifying antirheumatic drugs (csDMARDs) such as methotrexate (MTX) and the growing availability of biologic DMARDs (bDMARDs) have led to improved clinical outcomes [2–4]. In addition, the treat-to-target approach has been adopted in clinical practice and incorporated into international recommendations [5]. 

Etanercept, a tumor necrosis factor (TNF-)inhibitor, is one of the most widely used bDMARDs for non-systemic JIA [6–8]. Commonly, etanercept is started at the labelled dose of 0.8 mg/kg/week (max. 50 mg/week) [9]. Higher doses (up to 1.6 mg/kg/week, max. 50 mg/week) are used off-label in clinical practice if the labelled dose is ineffective [10]. Even though data from Canada and the USA have shown that approximately 10% of JIA-patients treated with etanercept received doses > 40% above the labelled dose, the effects of such off-label high-dose etanercept treatment, including the efficacy and safety profile, are not sufficiently known [10, 11]. 

Therefore, our primary objective was to provide an in-depth description of the clinical course of non-systemic JIA-patients receiving high-dose etanercept as part of the BeSt for Kids trial [12]. As a comparison group, we present the same data of patients who did not escalate to high-dose etanercept despite eligibility according to trial-protocol (protocol deviations).

## Methods

### Trial design

The BeSt for Kids (Dutch acronym for ‘treatment strategies for children’) study was described extensively elsewhere [12]. In short, this Dutch multi-centre trial evaluated three different treat-to-target regimens. Enrolment was from October 2009 to April 2014. It included 92 DMARD-naïve patients aged 2–16 years with new-onset oligoarticular JIA (*n* = 11), RF-negative polyarticular JIA (*n* = 73) or juvenile psoriatic arthritis (*n* = 8). Exclusion criteria comprised, among others, symptom duration ≥ 18 months, rheumatoid factor (RF) positivity and uveitis at enrolment. Please note that, due to the timing of patient recruitment right after diagnosis and efforts to start treatment early, oligoarticular JIA in this study comprises both persistent (≤ 4 joints affected in later progression) and extended oligoarthritis (> 4 joints affected in later progression).

Patients were randomised by variable block, stratified per centre and per oligoarticular or polyarticular disease, into three arms (1:1:1) with different initial treatments: (1) sequential DMARD-monotherapy (sulfasalazine or MTX), (2) combination-therapy of MTX and 6 weeks prednisolone, and (3) combination therapy of MTX and etanercept. Follow-up visits were planned every 3 months for two years. Median follow-up was 24.6 months.

**Fig. 1:**
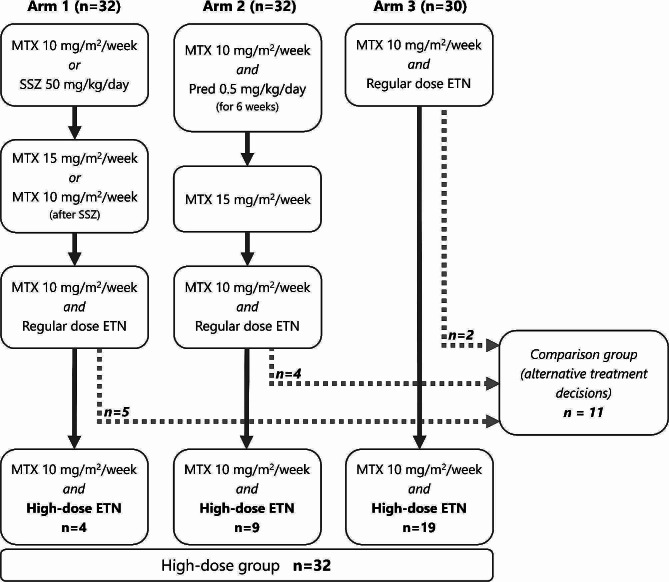
Illustration of patient selection for current analyses based on a simplified flowchart of treatment steps in the BeSt for Kids trial **Legend** This simplified flowchart was designed to briefly illustrate patient numbers for current analyses. Exclusions and loss to follow-up are not shown. Please refer to the original BeSt for Kids report (including flowchart) for more details [12]. 12 patients were not included in current analyses since they already received the maximum etanercept dose of 50 mg per week based on their body-weight (2 patients in arm 1, 8 in arm 2 and 2 in arm 3) Abbreviations: MTX = methotrexate; mg = milligrams; SSZ = sulfasalazine; Pred = prednisolone; ETN = etanercept

The treatment target was defined by an adjusted ACRPedi50% at 3 months and, afterwards, by inactive disease in line with the Wallace 2004 criteria [13]. If not met, treatment was escalated according to the pre-specified treat-to-target protocol. In any treatment-arm patients could eventually escalate to high-dose etanercept (1.6 mg/kg/week, max 50 mg/week) alongside MTX (10 mg/m2/week; Fig. 1). High-dose etanercept was defined as escalation from the regular dose to a higher dose in line with this trial protocol. Due to the maximum absolute dose of 50 mg per week, the etanercept dose expressed in mg per kg bodyweight will be lower than 1.6 for patients weighing more than 31 kg.

### Patients studied

32 patients were escalated to high-dose etanercept after median 10 months (interquartile range (IQR) 7–16; ‘high-dose group’). Median etanercept dose at that point was 1.3 mg/kg/week (IQR 1.1–1.5).

11 other patients were eligible for high-dose etanercept at median 10 months (IQR 7–15), but did not proceed with this (‘comparison group’). These patients deviated from the trial-protocol through shared decision making with the treating paediatric rheumatologist. 10 of these patients continued etanercept in the regular dose, while one patient switched to infliximab. Reasons for not increasing the etanercept dose, as well as the alternative treatment decisions made, are described in more detail in Supplementary Table 1.

12 patients (two in arm 1, eight in arm 2 and two in arm 3) did not increase their etanercept dose as they already received the maximum of 50 mg per week based on their body-weight; these patients were *not* included in current analyses. For all patients that were included, bodyweight at the moment of eligibility for etanercept dose increase is presented in Supplementary Fig. 1.

### Outcomes

Outcomes of interest were disease-activity measured by the JADAS-10 and its individual components [14], pain-intensity and functional impairments. A 0–100 mm visual analogue scale (VAS) was used to quantify pain intensity over the last 7 days, which was estimated by the parents for patients aged < 12 years, as described previously [15]. The number of active joints at each visit was assessed by a physician or physiotherapist blinded to treatment allocation (single-blinded study design). Functional impairments were assessed using the Childhood Health Assessment Questionnaire(CHAQ), ranging 0–3 with higher scores representing worse functioning [16]. 

In addition, the percentage of patients with inactive disease 6 months after eligibility for etanercept dose increase was calculated [15]. Inactive disease was defined by the Wallace 2004 criteria adjusted by physician’s global assessments < 10 mm indicating no active disease [12, 13]. The percentage of patients who subsequently lost this inactive disease criterium was also assessed.

### Statistical analyses

Analyses in this study are descriptive. Direct statistical comparisons between the high-dose and comparison group were not performed since the trial was not powered accordingly [12]. 

Medians of clinical parameters were plotted from the moment of eligibility for etanercept dose increase onwards. The Wilcoxon rank-sum test was used to calculate whether clinical parameters had changed statistically significantly at 3 months after eligibility for etanercept dose increase. The rate of non-severe adverse events (AEs) was calculated per group by dividing the number of AEs registered *after* etanercept dose increase by the number of subsequent patient years follow-up. Moreover, the AE rate per patient year was calculated for the period *until* the moment of eligibility for etanercept dose increase.

In order to filter out potential treatment effects of bDMARD switching (rather than maintaining the etanercept dose), analyses were repeated after excluding patients in the comparison group who switched to another bDMARD.

IBM SPSS v29 was used. Two-sided *p*-values < 0.05 were considered statistically significant.

## Results

### Patients

Patient characteristics at inclusion and at the moment of eligibility for etanercept dose-increase are presented in Table 1. In the high-dose group, median age was 6 years at inclusion (IQR 4–10) and 69% were girls. In the comparison group, median age was 8 years at inclusion (IQR 6–9) and 73% were girls. The comparison group had a higher number of actively inflamed joints at inclusion (median (IQR) 11 (8–18) compared to 7 (5–11) in the high-dose group, (*p* = 0.022), otherwise clinical parameters were generally similar in both groups.

**Table 1:** Characteristics of patients in the high-dose group and the comparison group at inclusion and at the moment of eligibility for high-dose etanercept

	At inclusion			At eligibility for etanercept dose increase		
	High-dose ETN group (*n* = 32)	Comparison group (*n* = 11)	*p*	High-dose ETN group (*n* = 32)	Comparison group (*n* = 11)	*p*
Time from inclusion in months, median (IQR)	0	0	–	10 (7–16)	10 (7–15)	0.98
Age, median (IQR)	6 (4–10)	8 (6–9)	0.61	*–*	*–*	
Female, n (%)	22 (69)	8 (73)	1.00	*–*	*–*	
JADAS-10, median (IQR)	18 (15–22)	20 (17–22)	0.36	7 (4–10)	9 (2–10)	0.92
VAS patient/parent, median (IQR)	63 (50–74)	66 (37–75)	0.94	39 (11–56)	38 (6–61)	1.00
VAS physician, median (IQR)	51 (41–58)	47 (40–65)	0.92	12 (2–18)	16 (8–31)	0.50
Number of active joints, median (IQR)	7 (5–11)	11 (8–18)	0.022	2 (0–4)	2 (1–2)	0.89
ESR in mm/hour, median (IQR)	9 (5–25)	8 (2–19)	0.65	6 (2–9)	7 (4–14)	0.54
VAS pain, median (IQR)	63 (49–72)	69 (54–80)	0.37	36 (9–62)	22 (6–68)	0.79
CHAQ, median (IQR)	1.07 (0.75–1.63)	1.50 (0.63–1.88)	0.39	0.63 (0.38–1.25)	0.69 (0.00–1.75)	0.99

**Legend**

Abbreviations: IQR = interquartile range; JADAS = Juvenile Arthritis Disease Activity Score; VAS = visual analogue scale; ESR = erythrocyte sedimentation rate;

mm = millimetres; CHAQ = childhood health assessment questionnaire

### Clinical parameters over time

**Fig. 2:**
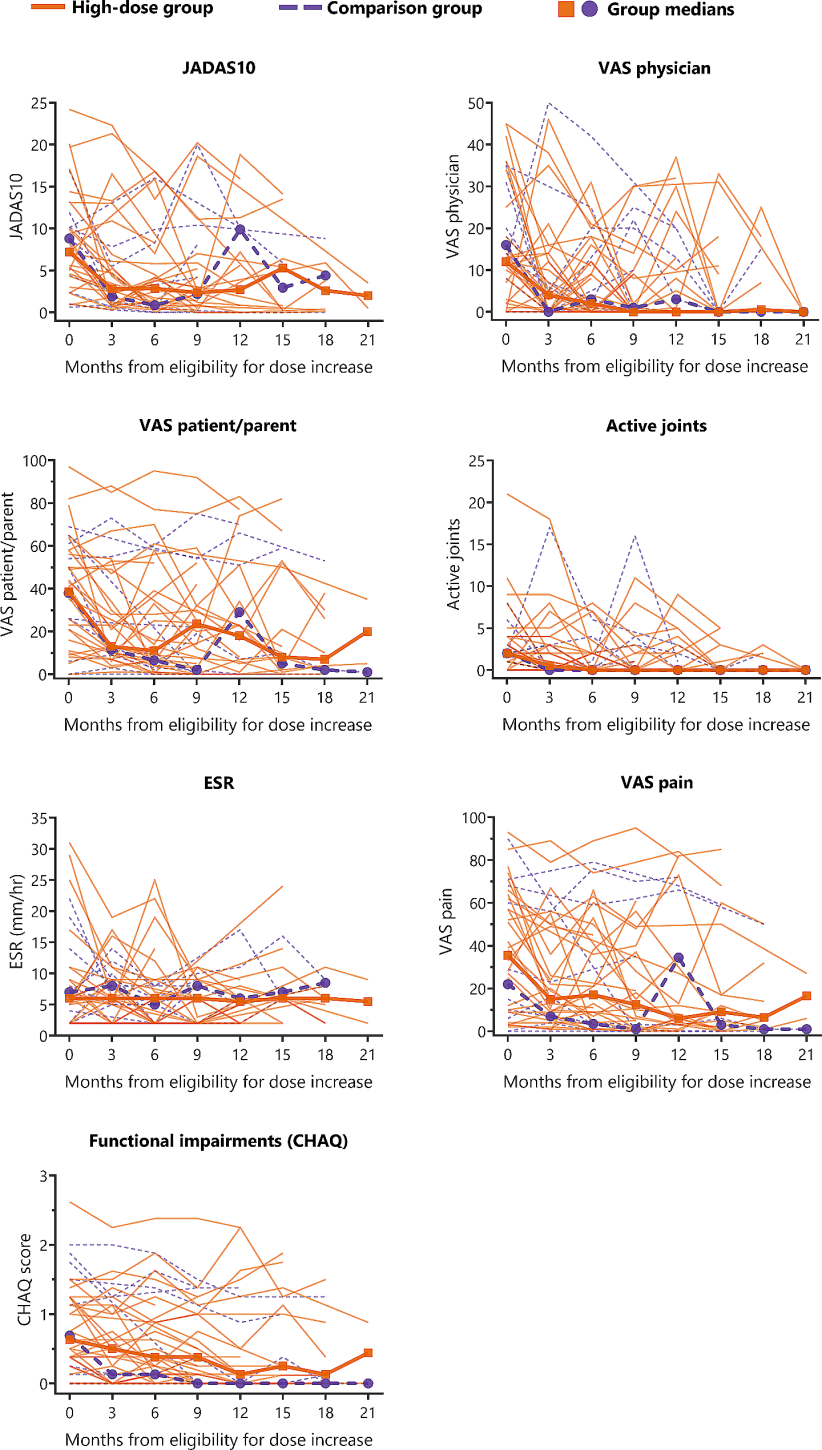
Clinical parameters over time from the moment of eligibility for etanercept dose increase for both the high-dose and the comparison group **Legend** Lines represent individual patients; squares and dots represent the group median Timepoint zero represents the moment of eligibility for etanercept dose increase, which is *not* equivalent to the baseline visit of the BeSt for Kids trial and may differ from patient to patient Abbreviations: JADAS = Juvenile Arthritis Disease Activity Score; VAS = visual analogue scale; CHAQ = childhood health assessment questionnaire; ESR = erythrocyte sedimentation rate

Follow-up was up to 2 years from baseline; median follow-up was 24.6 months. Clinical parameters over time from the moment of eligibility for etanercept dose increase are presented in Fig. 2. Overall, the clinical course seemed similar in both groups.

In the high-dose group, clinical measures of disease-activity improved largely within 3 months: median JADAS10 from 7.2 to 2.8 (*p* = 0.008), VAS-physician from 12 to 4 (*p* = 0.022), VAS-patient/parent from 38.5 to 13 (*p* = 0.003), VAS pain from 35.5 to 15 (*p* = 0.030), the number of active joints from 2 to 0.5 (*p* = 0.12) and functional status (CHAQ-score) from 0.63 to 0.50 (*p* = 0.047), while ESR remained stable (from 6 to 6; *p* = 0.32).

In the comparison group, a comparable pattern of clinical parameters over time was observed. After 3 months median JADAS10 improved from 8.8 to 1.9 (*p* = 0.017), VAS-physician from 16 to 0 (*p* = 0.24), VAS-patient/parent from 38 to 11.5 (*p* = 0.93), VAS pain from 22 to 7 (*p* = 0.67), the number of active joints from 2 to 0 (*p* = 0.29) and functional status (CHAQ-score) from 0.69 to 0.13 (*p* = 0.41), while ESR remained stable (from 7 to 8; *p* = 0.72).

Although in broad terms the clinical course appeared similar in both groups, at the 12 months timepoint specifically median JADAS10 and VAS pain were numerically lower (better) in the high-dose than in the comparison group (Fig. 2). However, at this point in time sample size was low (*n* = 11 in the high-dose group and *n* = 5 in the comparison group) and no direct statistical comparison was made.

### Inactive disease

In both the high-dose and the comparison group the percentage of patients with inactive disease 6 months after eligibility for dose-increase was 56%.

Loss of inactive disease criteria after eligibility for high dose etanercept occurred in 8 patients in the high-dose group (25%) and in 3 patients (27%) in the comparison group.

### Adverse events

No severe adverse events (SAEs) were recorded after etanercept dose-increase. In the comparison group there were 2 SAEs consisting of hospital admissions. One of these concerned supportive care for gastroenteritis. The other admission was for precautionary intravenous antiviral treatment due to mildly increased liver-enzymes together with a varicella infection (which can cause hepatitis in immunocompromised patients, but in this case the patient recovered without complications).

**Fig. 3:**
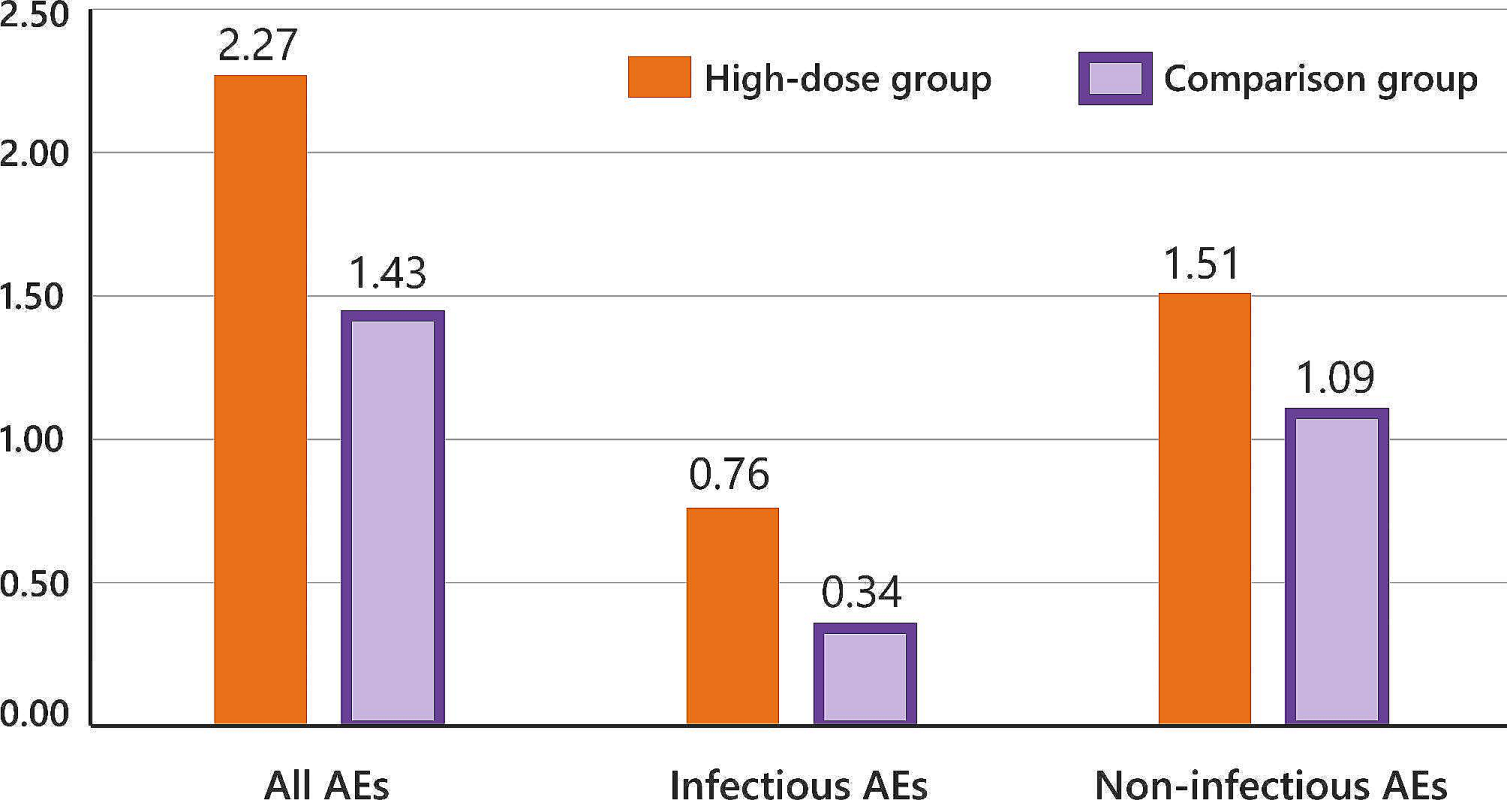
Adverse events in both the high-dose and the comparison group, expressed in rates per patient year following the moment of eligibility for etanercept dose increase **Legend** AEs = non-severe adverse events

Non-severe adverse events (AEs) are summarised in Fig. 3 and presented in more detail in Supplementary Table 2. In the high-dose group, 18 out of 32 patients (56%) experienced 26 infectious AEs; 26 patients (81%) experienced 78 AEs of any sort. In the comparison group, 4 out of 11 patients (36%) experienced 4 infectious AEs; 6 patients (55%) experienced 17 AEs of any sort. Median time from eligibility for dose-increase until occurrence of the AE appeared similar in both groups (median 6 months (IQR 3–9) for AEs in the high-dose group; 7 months (IQR 4–12) for AEs in the comparison group). The rate of infectious AEs per patient year following eligibility for etanercept dose increase was 0.76 in the high-dose group and 0.34 in the comparison group. For AEs of any sort, rates were 2.27 and 1.43 per visit, respectively. Thus, AEs were numerically more frequent in the high-dose than in the comparison group.

### Additional analyses

Findings were similar when one patient who switched to infliximab was excluded from the comparison group (Supplementary Figs. 2 and 3).

Next, we assessed AEs *until* eligibility for high-dose etanercept (Supplementary Fig. 4 and Supplementary Table 3). During this period, the overall rate of AEs per patient year was 3.26 in the high-dose group and 2.51 in the comparison group. Thus, the frequency of AEs was, numerically, already somewhat higher in the high-dose than in the comparison group before eligibility for etanercept dose-increase occurred.

## Discussion

Although high-dose etanercept is used off-label in JIA-patients in clinical practice, supporting evidence is lacking. Therefore, we conducted a post-hoc analysis of the BeSt for Kids trial describing the clinical course of JIA-patients who received high-dose etanercept and of those who did not receive high-dose etanercept despite eligibility according to trial protocol. Parameters of disease-activity and the disease-burden developed largely similarly over time in both groups. No SAEs were seen after escalation to high-dose etanercept. Non-severe AEs were numerically more frequent in the high-dose than in the comparison group.

The question whether higher doses of etanercept can contribute to reaching treatment goals in JIA is highly relevant. Adequate treatment can contribute to improved quality of life for individual patients and parents [17]. In addition, there may be important social benefits such as reducing caregiver work-productivity loss [17, 18]. On the other hand, dose increase may have disadvantages such as increased costs and, hypothetically, dose-dependent side-effects [19] which are desired to be in proportion to the benefits of the treatment. If this is not the case, other strategies such as switching to another bDMARD might be more appropriate.

Nevertheless, literature on this topic is scarce. Takei et al. reported 8 JIA-patients receiving high-dose etanercept but lack a comparison group [11]. Another study found no significant differences regarding clinical outcomes and AEs between JIA-patients who were escalated from regular to high-dose bDMARDs on the one hand, and bDMARD-switchers on the other hand, but did not report specifically on the 14 patients who escalated to high-dose etanercept [10]. Our study adds to this scarce evidence by describing clinical outcomes and AEs in the largest-to-date group of JIA-patients who were escalated to high-dose etanercept, and in a relevant comparison group.

This study has several limitations, including its small group size and descriptive nature. In addition, escalating from regular to high-dose etanercept was not randomised and blinded. We observed similar clinical improvements in both groups, even though treatment was not changed in most patients in the comparison group. It is possible that the etanercept dose was not increased in the comparison group because further clinical improvement was expected by the paediatric rheumatologist or by the patient/parents. This would be indicative of (unmeasured) confounding by indication, which may lead to false-negative findings. On the other hand, due to the lack of randomisation and blinding, one may have expected a placebo response to increasing the etanercept-dose. Still, the clinical course was similar in both groups. Moreover, due to the open label design, patients and their parents knew when their etanercept dose was increased which may theoretically have led to more alertness to adverse events.

Furthermore, it is unknown whether the numerically higher rate of adverse events observed in this study is causally related to higher etanercept dosage. Exploring this further, we calculated rates of non-severe AEs per visit *until* eligibility for high-dose etanercept in both groups, and found that the frequency of AEs was, numerically, already somewhat higher in the high-dose than in the comparison group. This poses an additional challenge to interpretation of the data on AEs. Possibly, patients in the high-dose group may have been inherently more prone to the occurrence of AEs due to (unknown) confounders. For example, younger children may be more prone to AEs when using etanercept and median age was numerically lower in the high-dose than in the comparison group (median difference of 2 years, which was not statistically significant).

Altogether, these results should be interpreted with caution. Selection bias and confounding by indication should be considered as influencing factors. Future, preferably randomized studies would be needed to obtain higher level evidence.

Lastly, we would like to point out that etanercept in this study was given alongside 10 mg/m^2^ MTX per week. The recommended dose for MTX in JIA-patients is 10 to 15 mg/m^2^. It was deemed appropriate to dose MTX in the low-normal range given the context of treatment-to-target and tightly scheduled follow-up, allowing swift access to combination therapy. We acknowledge that in current times, considering current consensus and guidelines for JIA treatment [20, 21], the higher MTX dose of 15 mg/m^2^ could be preferred – also alongside etanercept.

## Conclusions

In conclusion, escalation to high-dose etanercept in JIA-patients who were treated to target was generally followed by meaningful clinical improvement. However, similar improvements were observed in a smaller comparison group who did not escalate to high-dose etanercept. No SAEs were seen after escalation to high-dose etanercept. We advocate larger, randomised studies of high versus regular dose etanercept to provide high level evidence on efficacy and safety.

### Electronic supplementary material

Below is the link to the electronic supplementary material.

Supplementary Material 1

Supplementary Material 2

Supplementary Material 3

## Data Availability

Data are available from the corresponding author (e-mail; B.T.van_Dijk@lumc.nl) upon reasonable request.

## Abbreviations

AE: Adverse event
bDMARD: Biologic DMARDs
BeSt: Behandelstrategieën (Dutch for ‘treatment strategies’)
CHAQ: Childhood Health Assessment Questionnaire
csDMARD: Conventional synthetic DMARD
DMARD: Disease-modifying anti-rheumatic drugs
ESR: Erythrocyte sedimentation rate
ETN: Etanercept
IBM SPSS v29: International Business Machines Corporation Statistical Package for the Social Sciences version 29
IQR: Interquartile range
JADAS: Juvenile Arthritis Disease Activity Score
JIA: Juvenile idiopathic arthritis
LUMC: Leiden University Medical Centre
mg: Milligrams
mm: Millimeters
MTX: Methotrexate
Pred: Prednisolone
RF: Rheumatoid factor
SAE: Severe adverse event
SSZ: Sulfasalazine
TNF: Tumor necrosis factor
VAS: Visual analogue scale
