# Supplementary material for: Increasing the etanercept dose in a treat-to-target approach in juvenile idiopathic arthritis: does it help to reach the target? A post-hoc analysis of the BeSt for Kids randomised clinical trial
Source: Pediatr Rheumatol Online J. 2024 May 10;22:53. doi: 10.1186/s12969-024-00989-x (PMC11084083; doi:10.1186/s12969-024-00989-x)
Supplement: Supplementary file 2 — Supplementary Material 2 [file 12969_2024_989_MOESM2_ESM.docx]

**Supplementary File**

**Supplementary Table 1. Reasons for not escalating to high-dose etanercept and alternative treatment decisions in the comparison group (n=11)**

| **n** | **Reason** | **Alternative decision** |
| --- | --- | --- |
| 3 | Lack of therapeutic compliance | No change in treatment (watchful waiting) |
| 2 | The clinical interpretation of the physician was that the patient was in inactive disease, since only one joint was dubiously active while all other parameters were favorable |  |
| 1 | The clinical interpretation of the physician was that the patient was in inactive disease, since there was only a borderline increased ESR while no joints were active and the physician’s global assessment was zero |  |
| 1 | Presence of an intercurrent infection and only dubiously active synovitis |  |
| 2 | The patient stopped MTX on his/her own accord | Restart MTX |
| 1 | Presence of an intercurrent infection and a history of worsening of the symptoms after stopping NSAIDs | Addition of NSAID |
| 1 | Needle phobia | Switch to infliximab |

**Legend**

Abbreviations: ESR = erythrocyte sedimentation rate; MTX = methotrexate; NSAID = non-steroidal anti-inflammatory drug

**Supplementary Table 2. Summary of non-severe adverse events recorded after the moment of eligibility for etanercept dose increase in both the high-dose and the comparison group, until the end of follow-up**

|  | **Number of AEs** | | **Patients with ≥1 AE (% of group total)** | | **AE rate per patient year** | |
| --- | --- | --- | --- | --- | --- | --- |
|  | *High-dose group*  *(n=32)* | *Comparison group*  *(n=11)* | *High-dose group*  *(n=32)* | *Comparison group*  *(n=11)* | *High-dose group*  *(n=32)* | *Comparison group*  *(n=11)* |
| **Infectious AEs** | **26** | **4** | **18 (56)** | **4 (36)** | **0.76** | **0.34** |
| Upper respiratory tract infection | 7 | 1 | 6 (19) | 1 (9) | 0.20 | 0.08 |
| Skin / mucosal infections | 3 | 1 | 3 (9) | 1 (9) | 0.09 | 0.08 |
| Gastroenteritis | 2 | 0 | 2 (6) | 0 (0) | 0.06 | 0 |
| Urinary tract infection | 2 | 1 | 2 (6) | 1 (9) | 0.06 | 0.08 |
| Other | 12 | 1 | 11 (34) | 1 (9) | 0.35 | 0.08 |
| **Other AEs** | **52** | **13** | **20 (63)** | **8 (73)** | **1.51** | **1.09** |
| Nausea or abdominal pain | 12 | 0 | 8 (25) | 0 (0) | 0.35 | 0 |
| Non-infectious skin abnormalities | 8 | 2 | 6 (19) | 1 (9) | 0.23 | 0.17 |
| General malaise | 3 | 1 | 3 (9) | 1 (9) | 0.09 | 0.08 |
| Liver enzyme abnormalities | 3 | 5 | 3 (9) | 3 (27) | 0.09 | 0.42 |
| Coughing | 3 | 0 | 2 (6) | 0 (0) | 0.09 | 0 |
| Headache | 2 | 0 | 1 (3) | 0 (0) | 0.06 | 0 |
| Asthma | 1 | 0 | 1 (3) | 0 (0) | 0.03 | 0 |
| Other | 20 | 5 | 12 (38) | 4 (36) | 0.58 | 0.42 |
| **Any AE** | **78** | **17** | **26 (81)** | **6 (55)** | **2.27** | **1.43** |

**Legend**

AE = non-severe adverse event

**Supplementary Table 3. Summary of non-severe adverse events recorded *until* the moment of eligibility for etanercept dose increase in both the high-dose and the comparison group**

|  | **Number of AEs** | | **Patients with ≥1 AE (% of group total)** | | **AE rate per patient year** | |
| --- | --- | --- | --- | --- | --- | --- |
|  | *High-dose group*  *(n=32)* | *Comparison group*  *(n=11)* | *High-dose group*  *(n=32)* | *Comparison group*  *(n=11)* | *High-dose group*  *(n=32)* | *Comparison group*  *(n=11)* |
| **Infectious AEs** | **29** | **5** | **15 (47)** | **3 (27)** | **0.94** | **0.48** |
| Upper respiratory tract infection | 18 | 1 | 14 (44) | 1 (9) | 0.58 | 0.10 |
| Skin / mucosal infections | 2 | 2 | 2 (6) | 1 (9) | 0.06 | 0.19 |
| Gastroenteritis | 2 | 1 | 2 (6) | 1 (9) | 0.06 | 0.10 |
| Urinary tract infection | 1 | 0 | 1 (3) | 0 (0) | 0.03 | 0 |
| Other | 6 | 1 | 4 (13) | 1 (9) | 0.19 | 0.10 |
| **Other AEs** | **72** | **21** | **24 (75)** | **7 (64)** | **2.33** | **2.02** |
| Nausea or abdominal pain | 23 | 4 | 16 (50) | 3 (27) | 0.74 | 0.39 |
| Non-infectious skin abnormalities | 10 | 4 | 8 (25) | 3 (27) | 0.32 | 0.39 |
| General malaise | 6 | 1 | 4 (13) | 1 (9) | 0.19 | 0.10 |
| Liver enzyme abnormalities | 8 | 2 | 6 (19) | 2 (18) | 0.26 | 0.19 |
| Coughing | 0 | 0 | 0 (0) | 0 (0) | 0 | 0 |
| Headache | 3 | 0 | 2 (6) | 0 (0) | 0.10 | 0 |
| Asthma | 0 | 0 | 0 (0) | 0 (0) | 0 | 0 |
| Other | 22 | 10 | 15 (47) | 6 (55) | 0.71 | 0.96 |
| **Any AE** | **101** | **26** | **26 (81)** | **9 (82)** | **3.26** | **2.51** |

**Legend**

AE = non-severe adverse event

**Supplementary Figure 1. Bodyweight at the moment of eligibility for etanercept dose increase in both the high-dose and the comparison group**

**
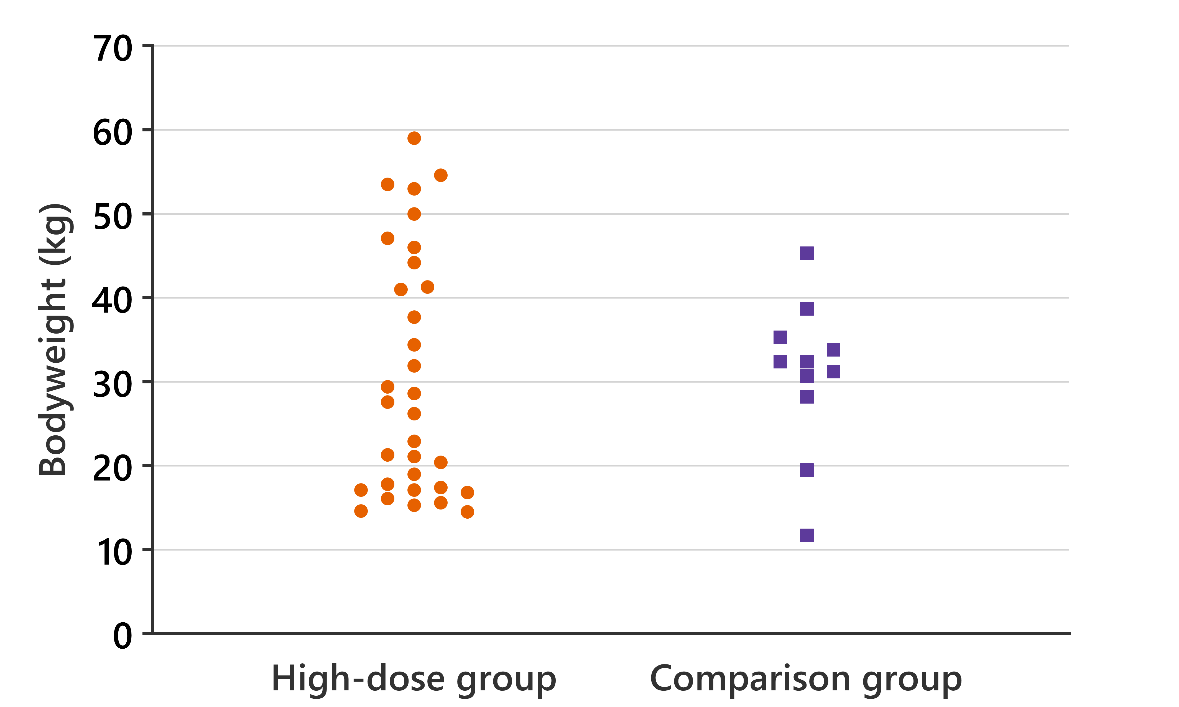
**

**Legend**

Abbreviations: kg = kilograms

**Supplementary Figure 2. Clinical parameters over time from the moment of eligibility for etanercept dose increase for both the high-dose and the comparison group, after excluding one patient who switched to infliximab**


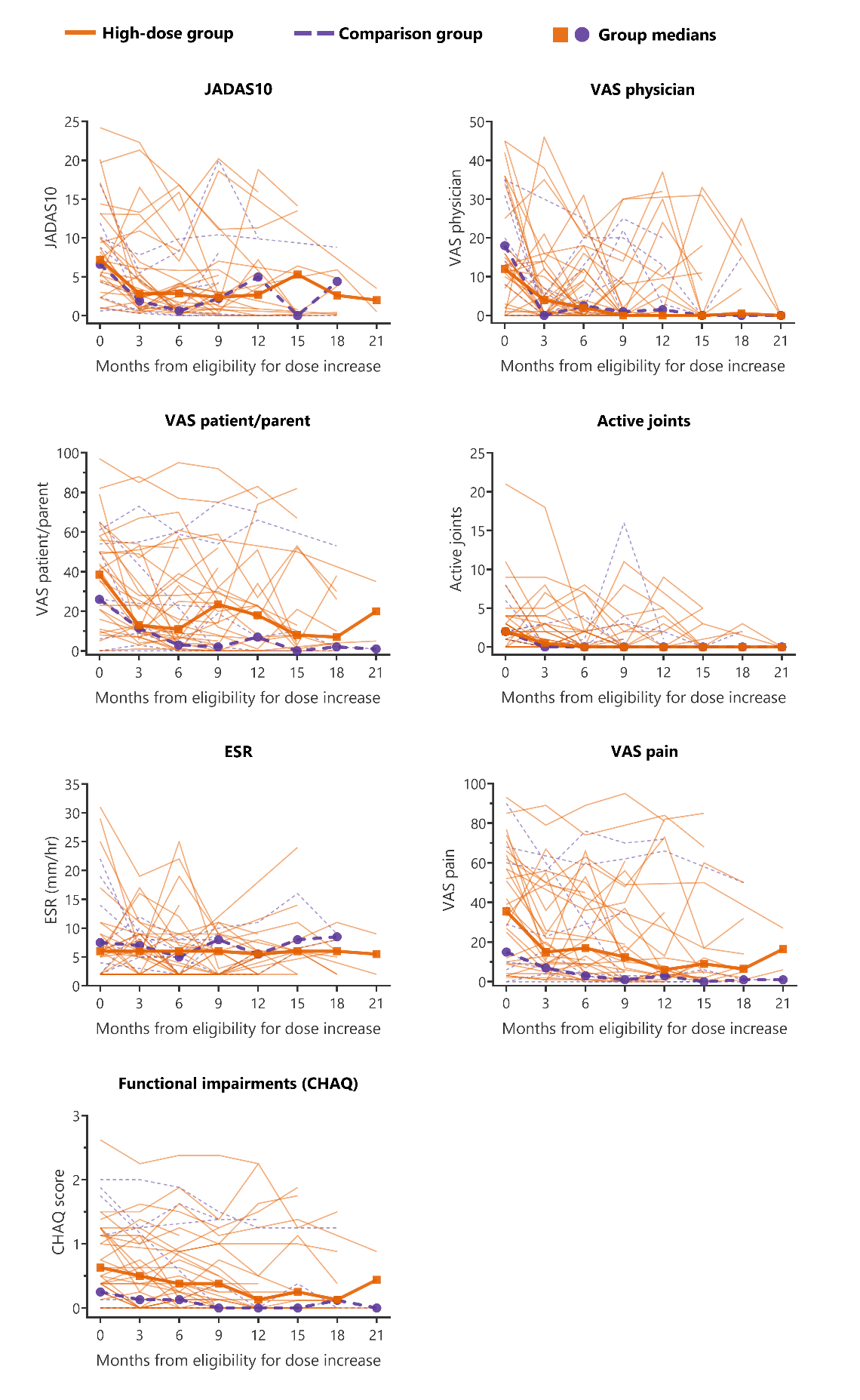


**Legend *(continues on next page)***

Lines represent individual patients; squares and dots represent the group moment.

Timepoint zero represents the moment of eligibility for etanercept dose increase, which is *not* equivalent to the baseline visit of the BeSt4Kids trial and may differ from patient to patient.

Abbreviations: JADAS = Juvenile Arthritis Disease Activity Score; VAS = visual analogue scale;
ESR = erythrocyte sedimentation rate; CHAQ = childhood health assessment questionnaire

**Supplementary Figure 3.** **Adverse events, expressed in rates per patient year following the moment of eligibility for etanercept dose increase, after excluding one patient who switched to infliximab**


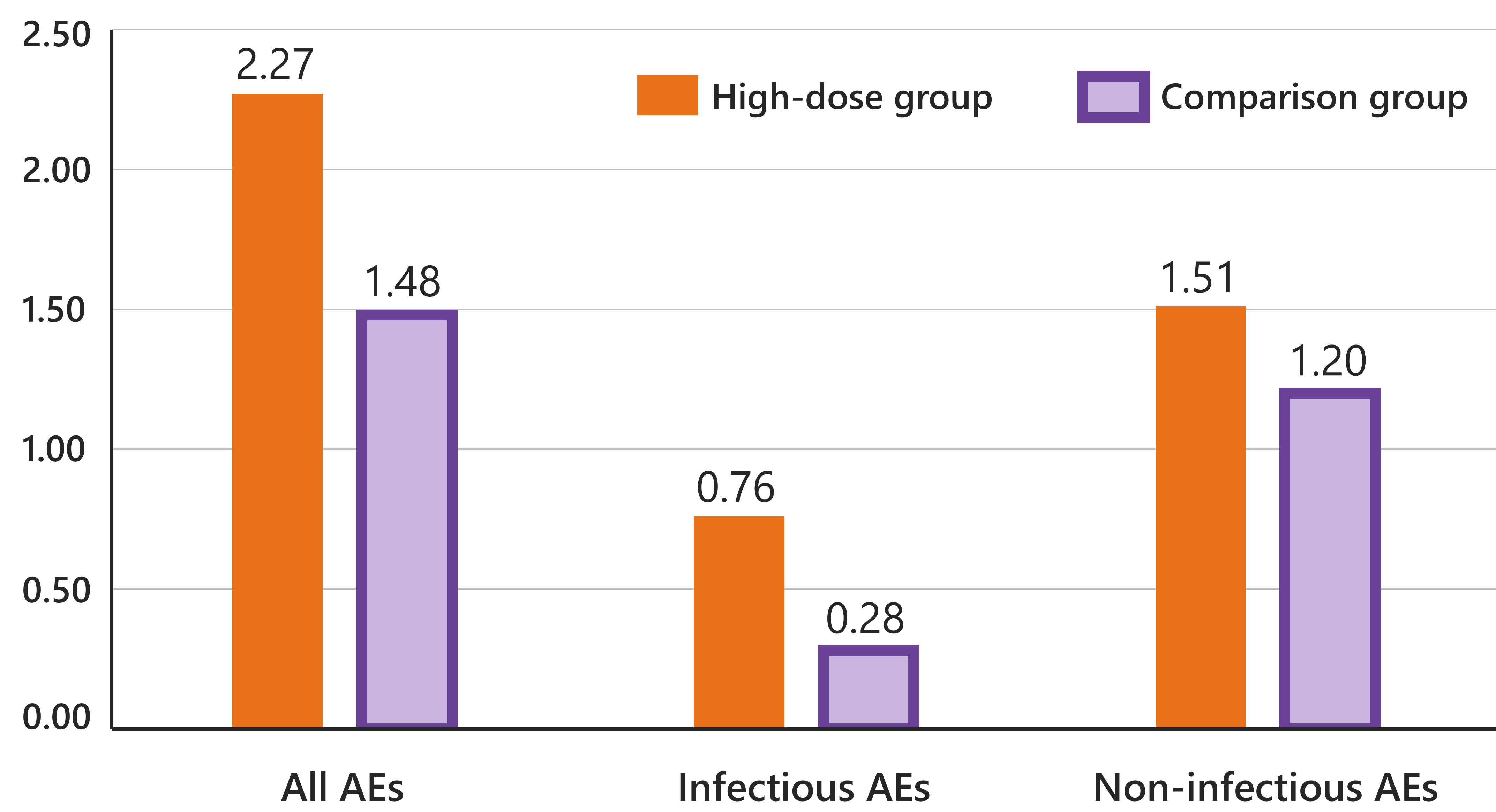


**Legend**

AEs = adverse events

**Supplementary Figure 4. Adverse events, expressed in rates per patient year *until* the moment of eligibility for etanercept dose increase**


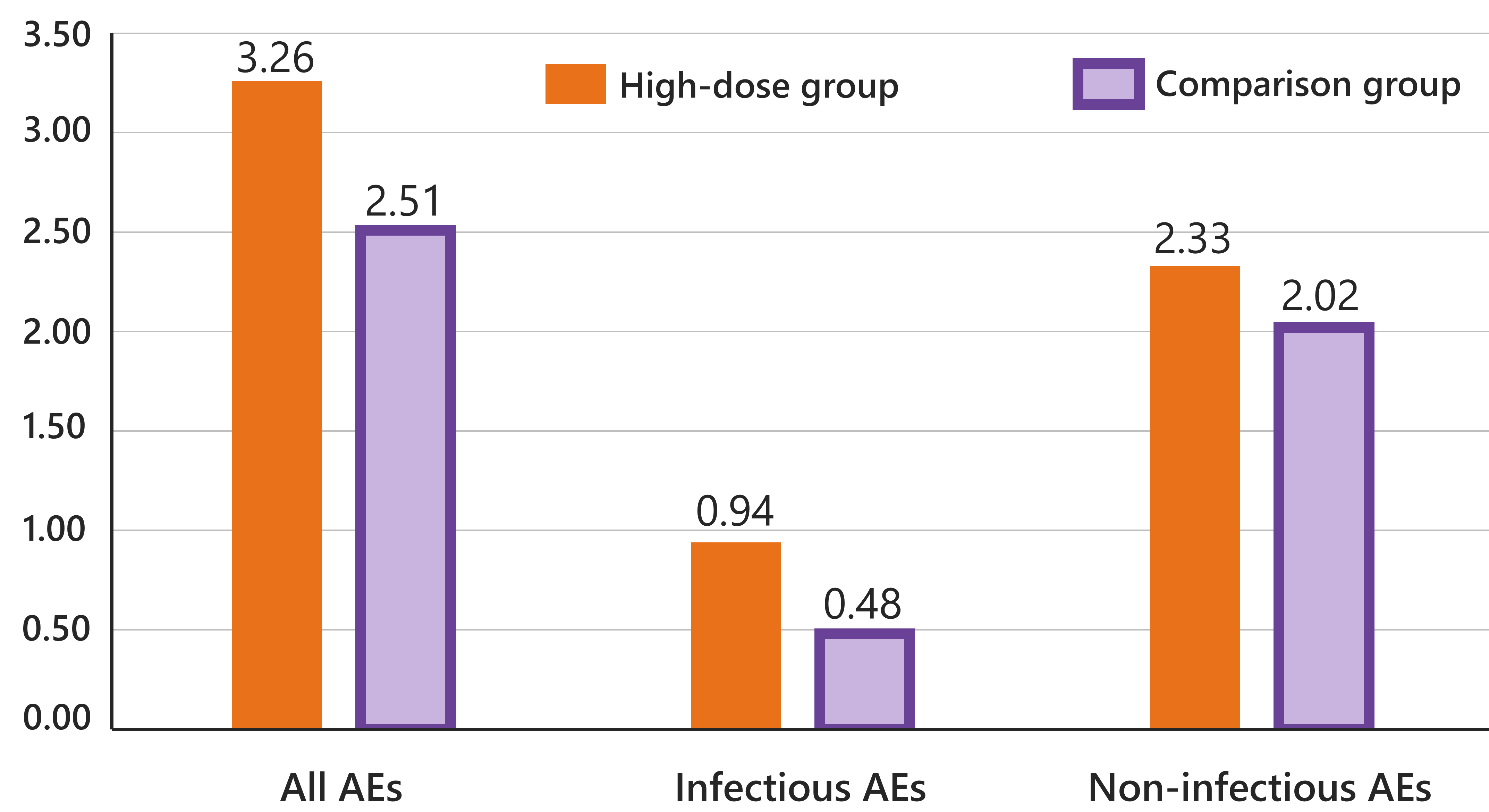


**Legend**

AEs = adverse events
